# Prevalence and Factors Associated with Microbial and Methicillin-Resistant Staphylococcal Contamination of Smartphones Among Medical Students at Six Universities in Klang Valley, Malaysia

**DOI:** 10.3390/antibiotics15100952

**Published:** 2026-09-24

**Authors:** Cin Dee Lee, San Wei Hii, Xin Ker Quek, Jia Xuan Tan, Rachel Yee Chia Wong, Eddy Seong Guan Cheah, Yun Fong Ngeow, Hien Fuh Ng

**Affiliations:** 1 M. Kandiah Faculty of Medicine and Health Sciences, Sungai Long Campus, Universiti Tunku Abdul Rahman (UTAR), Kajang 43000, Selangor, Malaysia; cdlee@utar.edu.my (C.D.L.); thdr.sanwei@1utar.my (S.W.H.); xinkerquek@1utar.my (X.K.Q.); jiaxuan0307@1utar.my (J.X.T.); rachelrwyc@1utar.my (R.Y.C.W.); ngeowyf@utar.edu.my (Y.F.N.); 2 Centre for Foundation Studies, Sungai Long Campus, Universiti Tunku Abdul Rahman (UTAR), Kajang 43000, Selangor, Malaysia; 3 Faculty of Science, Kampar Campus, Universiti Tunku Abdul Rahman (UTAR), Kampar 31900, Perak, Malaysia; cheahsg@utar.edu.my

**Keywords:** methicillin-resistant staphylococci, fomite, total viable count, contamination, risk factors, health promotion

## Abstract

Background/Objectives: Smartphones used by medical students may serve as reservoirs for microbial contamination and contribute to the transmission of healthcare-associated microorganisms. This study aimed to determine the level of microbial contamination of smartphones, prevalence of methicillin-resistant *Staphylococcus* species (MRSS), and associated risk factors among undergraduate medical students in Klang Valley, Malaysia. Methods: An analytical cross-sectional study was conducted among students from three public and three private universities using convenience and snowball sampling. Demographic, behavioural, and environmental data were collected via an online questionnaire. Smartphone swabs were cultured to determine total viable count (TVC). MRSS were identified using enrichment and selective culture, Gram staining, 16S rRNA PCR-sequencing, and cefoxitin disc diffusion. Descriptive statistics, Fisher’s exact test, and multivariable binary logistic regression were performed. Results: A total of 148 smartphones were analysed. High microbial contamination (≥13 CFU per smartphone sampling unit, 75th percentile of TVC) was detected in 38 (25.7%) smartphones, while MRSS were recovered from 9 (6.1%) smartphones; all were methicillin-resistant coagulase-negative staphylococci: *Staphylococcus epidermidis*, *Staphylococcus haemolyticus*, and *Staphylococcus ureilyticus*. Male students (adjusted odds ratio [AOR] = 2.497, *p* = 0.025) and students from public universities (AOR = 2.730, *p* = 0.016) were more likely to have highly contaminated smartphones. MRSS contamination was associated with high microbial contamination (OR = 12.194, *p* = 0.001) and those living with elderly individuals (OR = 6.304, *p* = 0.013). Following the study, participants received their culture results, smartphone hygiene education through a dedicated website, and alcohol swabs as part of a health promotion initiative. Conclusions: This study highlights smartphones as potential fomites for the transmission of antimicrobial-resistant staphylococci. Integrating microbiological surveillance with targeted smartphone hygiene promotion may help reduce microbial contamination and the potential for transmission in healthcare training environments.

## 1. Introduction

Antimicrobial resistance (AMR) is a major global public health challenge, with methicillin-resistant *Staphylococcus* species (MRSS) posing a significant threat because of their resistance to most β-lactam antibiotics and their ability to cause healthcare-associated infections. While methicillin-resistant *Staphylococcus aureus* (MRSA) has received considerable attention and is recognised by the World Health Organization as a high-priority pathogen [1], methicillin-resistant coagulase-negative staphylococci (MR-CoNS) are increasingly acknowledged as important opportunistic pathogens and reservoirs of antimicrobial resistance genes. As common colonisers of the human skin, CoNS, particularly *Staphylococcus epidermidis*, are frequently associated with device-related infections and infections among immunocompromised individuals [2]. Their presence on frequently touched surfaces may therefore contribute to the transmission of resistant microorganisms in both healthcare and community settings.

Smartphones have become indispensable tools in medical education and clinical practice. However, their frequent handling, prolonged use, and infrequent disinfection enable the accumulation and persistence of microorganisms, allowing them to serve as potential fomites. Nearly all smartphones used by healthcare workers have been reported to harbour potentially pathogenic bacteria, including MRSS, and may facilitate indirect transmission through repeated hand–device–face contact [3]. Despite increasing awareness of hand hygiene, routine cleaning of personal mobile devices remains largely overlooked in infection prevention practices, creating opportunities for bacterial persistence and dissemination.

Medical students represent an important population because they routinely transition between community, academic, and clinical environments. Their frequent smartphone use, combined with variable hand hygiene practices and increasing patient contact during clinical training, may increase the likelihood of microbial contamination and subsequent transmission of opportunistic pathogens, including antimicrobial-resistant bacteria. Nevertheless, evidence regarding smartphone contamination among Malaysian medical students remains limited. Existing studies have primarily focused on MRSA contamination [4,5] or nasal carriage of resistant bacteria [6,7], while molecular characterisation of MRSS contaminating personal devices has received little attention. In addition, behavioural and environmental factors, including smartphone disinfection practices, hand hygiene, living environment, and clinical exposure, remain insufficiently characterised.

This study aimed to determine the level of microbial contamination and the prevalence of MRSS contaminating smartphones among undergraduate medical students from public and private universities in Klang Valley, Malaysia. In addition, this study also aimed to identify demographic, behavioural, and environmental factors associated with microbial contamination and MRSS contamination. Beyond surveillance, the findings were translated into a health promotion initiative in which participants received their culture results, smartphone hygiene education through a dedicated website, and alcohol swabs to encourage regular smartphone disinfection. The findings provide baseline epidemiological evidence to support infection prevention strategies and reinforce the importance of integrating smartphone hygiene into health promotion programmes for future healthcare professionals, consistent with global efforts to reduce AMR transmission in healthcare settings [8].

## 2. Materials and Methods

### 2.1. Study Design and Participants

There are 16 universities offering the MBBS programme in Klang Valley. After excluding institutions whose clinical training facilities were located exclusively outside the region, as well as newly established medical programmes that had not yet progressed to the clinical years, five public and six private universities remained eligible for inclusion in the study. An analytical cross-sectional study was conducted between April and July 2026 among undergraduate medical students from six of these eligible universities in Klang Valley.

Participants were recruited using convenience and snowball sampling. Eligible participants were undergraduate MBBS students aged ≥ 18 years who owned a smartphone and provided informed consent. They were first approached through convenience sampling using student networks and university communication platforms. Participants were subsequently encouraged to introduce and refer other medical students who met the inclusion criteria, thereby facilitating additional recruitment through snowball sampling.

### 2.2. Questionnaire

Participants completed a structured online questionnaire assessing their demographic, behavioural, and environmental characteristics. The questionnaire was administered via Google Forms and comprised 37 questions (Supplementary Material S1).

### 2.3. Sample Collection

The entire front (screen) and back surfaces of each smartphone were sampled, with the back surface referring to either the phone itself or the external phone cover/case, where applicable. Sampling was performed using sterile swabs in 1 mL of liquid Amies transport medium (HiMedia Laboratories, India). The swabs were placed in an insulated box with ice packs for transport to the laboratory and were processed within 24 h of collection. The swab samples were vortexed for 10 s before inoculation onto the culture medium.

### 2.4. Total Viable Count (TVC)

For microbial enumeration, 50 µL of each specimen was inoculated onto plate count agar (Chemsol, Malaysia) and incubated aerobically at 35 °C for 48 h. Colonies were counted and expressed as colony-forming units (CFU).

### 2.5. Isolation and Characterisation of Methicillin-Resistant Staphylococci

An additional 50 µL of each specimen was enriched in 950 µL of tryptic soy broth (Liofilchem, Italy) at 35 °C with shaking at 200 rpm for 24 h. Subsequently, 50 µL of each enriched culture was plated onto HiCrome MRSA agar (HiMedia Laboratories) and incubated at 35 °C for 48 h. Green colonies were subcultured onto blood agar (Isolab, Malaysia) to obtain pure cultures, followed by Gram staining. Quality-control testing of HiCrome MRSA agar was performed using bacterial strains from our biobank, including positive controls comprising a clinical MRSA isolate and five MR-CoNS isolates (*Staphylococcus capitis*, *Staphylococcus haemolyticus*, *Staphylococcus cohnii*, *Staphylococcus warneri*, and *S. epidermidis*) previously recovered from healthy individuals [6]. Negative controls consisted of methicillin-susceptible *S. aureus* ATCC 29213 and *Escherichia coli* ATCC 25922.

Presumptive staphylococcal isolates were tested for methicillin resistance by cefoxitin disc diffusion according to EUCAST methodology and breakpoints [9]. Briefly, bacterial growth from a pure culture was emulsified in saline and adjusted to a 0.5 McFarland standard. The suspension was then evenly inoculated onto Mueller–Hinton agar (Isolab) using a sterile swab. A 30-µg cefoxitin disc was placed on the agar surface, and the plates were incubated at 35 °C for 20 h. *S. aureus* ATCC 29213 was used as the quality-control strain for disc diffusion.

Species identification was performed by bi-directional Sanger sequencing of the 16S rRNA gene and by comparing primer-trimmed sequences with sequences in the NCBI GenBank database (rRNA/ITS databases) using the Basic Local Alignment Search Tool (BLAST), as previously described [6,10]. Briefly, DNA was extracted from bacterial isolates using the boiling method. The 16S rRNA gene was amplified by PCR using the universal bacterial primers 27F (AGAGTTTGATCMTGGCTCAG) and 907R (CCGTCAATTCMTTTGAGTTT) [11] with GoTaq Green Master Mix (Promega, Wisc, USA). PCR amplification was performed with an initial denaturation at 95 °C for 5 min, followed by 30 cycles of denaturation at 95 °C for 1 min, annealing at 55 °C for 1 min, and extension at 72 °C for 1 min, with a final extension at 72 °C for 5 min.

### 2.6. Statistical Analysis

Data were analysed using IBM SPSS 31 or GraphPad Prisms 5. TVC values were dichotomised using the 75th percentile (≥13 CFU) to define high microbial contamination. Descriptive statistics were reported as frequencies and percentages. Associations between categorical variables and contamination outcomes (MRSS or high microbial contamination) were evaluated using Fisher’s exact test, with odds ratios (ORs) and 95% confidence intervals (CIs) reported. Variables with *p* < 0.25 in univariate analysis were included in multivariable binary logistic regression to identify factors independently associated with contamination outcomes. To maintain model stability and minimise overfitting, the number of predictors included was kept appropriate relative to the number of outcome events, following the general principle of at least 10 events per variable (EPV). Statistical significance was set at *p* < 0.05.

## 3. Results

### 3.1. Sampling

Three public and three private universities were included in this study, which represents 55% (6/11) of the eligible medical universities in Klang Valley.

A total of 148 medical students from these universities participated in this study, with 77 (52.0%) preclinical (Years 1 and 2) and 71 (48.0%) clinical students (Years 3, 4, and 5) (Table 1). All participants completed the questionnaire and smartphone swabbing procedures, resulting in a 100% completion rate with no missing data.

### 3.2. Descriptive Statistics

Table S1 summarises the demographic characteristics, smartphone-related behavioural practices, environmental exposures, and clinical-related characteristics of the study participants relevant to potential microbial contamination. The participants represented a typical undergraduate medical student population, with ages ranging from 19 to 26 years. Female students comprised the majority of participants (89/148, 60.1%).

Most participants demonstrated awareness regarding potential microbial contamination of smartphones, with 81.8% (121/148) reporting that smartphones could harbour microorganisms. The majority also reported having no current skin infections or open wounds (141/148, 95.3%). In terms of hygiene practices, regular use of hand sanitiser was reported by 115/148 (77.7%) participants.

Despite generally good hygiene awareness, several smartphone-handling behaviours associated with possible contamination were frequently reported. Smartphone use in the toilet was reported by 98/148 (66.2%) participants, while 87/148 (58.8%) reported placing their smartphones on toilet surfaces.

Smartphone cleaning frequency and methods varied among participants. More than half reported cleaning their smartphones within the previous week (83/148, 56.1%), including cleaning within the previous 24 h (26/148, 17.6%) and within the previous 2–7 days (57/148, 38.5%). However, less frequent cleaning practices were also observed, with 32/148 (21.6%) participants reporting cleaning within the previous month, 19/148 (12.8%) within the past year, and 14/148 (9.5%) reporting that they had never cleaned their smartphones.

Environmental exposure characteristics showed varying levels of potential microbial exposure. For example, 81.1% (120/148) reported not spending at least four hours with elderly individuals during the previous seven days. In contrast, more than half reported spending at least four hours with healthcare workers during the same period (76/148, 51.4%).

It should be noted that clinical exposure questions in the questionnaire (e.g., phone use during ward rounds or patient examinations and placing phones on hospital surfaces) were only applicable to clinical students who had commenced their clinical postings (*n* = 71) and were therefore only presented to this group in the online questionnaire. These questions were not applicable to preclinical students (*n* = 77). For the purpose of constructing contingency tables and performing statistical analyses, preclinical students were coded under the reference category representing no clinical exposure for each clinical-related variable (i.e., the perceived lower-risk category).

### 3.3. TVC and Prevalence of MRSS Contamination (All Identified as MR-CoNS)

TVC provided an estimation of the total microbial burden present on the smartphone surfaces. Among the 148 smartphone samples analysed, TVC values varied considerably, ranging from below the detection limit of the assay to 930 CFU per smartphone sampling unit. Most smartphones (*n* = 35) had microbial counts below the detection limit, whereas one smartphone recorded an exceptionally high microbial count of 930 CFU per smartphone sampling unit. The mean TVC was 20.6 CFU, while the median was much lower at 4 CFU, suggesting that the mean value was influenced by a small number of smartphones with very high microbial counts. The interquartile range was 12 CFU.

As there is currently no established microbiological cut-off value for defining high microbial contamination on smartphones, the 75th percentile of the observed TVC distribution (13 CFU) was used as an operational threshold in this study. Based on this classification, 38/148 smartphones (25.7%) were categorised as having high microbial contamination, while the remaining 110 smartphones (74.3%) were classified as having low-to-no microbial contamination. This categorisation was subsequently used to investigate whether high microbial contamination was associated with demographic, behavioural, and environmental factors.

Among the 148 smartphone samples analysed, 9 were positive for MRSS, giving an overall prevalence of 6.1% (95% CI: 2.8–11.2%). All MRSS isolates were identified as MR-CoNS (Table 2): six of them were *S. epidermidis*, two were *S. haemolyticus*, and one was *Staphylococcus ureilyticus*.

### 3.4. Inferential Analysis

The associations between selected demographic, behavioural, and environmental factors with MRSS contamination status were assessed using Fisher’s exact test (Table S2). Due to the small number of MRSS-positive cases (*n* = 9), multivariable logistic regression was not performed as it did not fulfil the 10-EPV rule. Living with elderly individuals was more frequently reported among participants with MRSS-positive smartphones (5/9, 55.6%) compared with those with MRSS-negative smartphones (23/139, 16.5%). This association was statistically significant (OR = 6.304, 95% CI: 1.572–25.282, *p* = 0.013). Interestingly, among the MRSS-positive samples, a higher proportion demonstrated high microbial contamination based on the TVC classification. Specifically, 7/9 (77.8%) MRSS-positive smartphones had high microbial contamination (≥75th percentile, ≥13 CFU) compared with 31/139 (22.3%) among MRSS-negative smartphones. Concordantly, Fisher’s exact test showed that high microbial contamination was significantly and positively associated with MRSS contamination (OR = 12.194, 95% CI: 2.410–61.704, *p* = 0.001).

To identify factors potentially associated with high microbial contamination of smartphones, Fisher’s exact test was first conducted (Table S3). Variables with a *p* < 0.25 in the univariate analysis were considered for inclusion in the multivariable binary logistic regression model. Five variables met this criterion (Table S3): sex (*p* = 0.012), knowledge that smartphones can carry microorganisms (*p* = 0.086), smartphone use during ward rounds (*p* = 0.185), staying with a healthcare worker(s) (*p* = 0.059), and type of university (*p* = 0.002). As all five variables were considered biologically plausible, the four variables with the smallest *p*-values were selected (Table 3).

The final model included 38 smartphones with high microbial contamination and four explanatory variables, giving an EPV of 9.5 (38 events/4 variables), which is roughly equal to 10. After adjusting for the selected variables, male sex and studying at a public university remained significantly associated with high microbial contamination of smartphones. Male participants had approximately 2.5 times higher odds of having high microbial contamination compared with female participants (AOR = 2.497, 95% CI: 1.120–5.568, *p* = 0.025). Similarly, students from public universities had approximately 2.7 times higher odds of high microbial contamination compared with students from private universities (AOR = 2.730, 95% CI: 1.206–6.177, *p* = 0.016).

### 3.5. Health Promotion

To translate the study findings into public health action, a health promotion campaign was conducted to raise awareness of smartphone microbial contamination, particularly MRSS, among medical students and the wider community. An educational website was developed as the main platform to communicate key findings, smartphone hygiene practices, and preventive measures in a user-friendly format (https://sites.google.com/1utar.my/chp-mrss-phonecontamination/findings). The website featured real microbiological demonstrations from this study, including comparisons of bacterial contamination between a smartphone and a toilet seat, as well as the reduction in bacterial growth following cleaning with a 70% alcohol swab. These visual demonstrations highlighted the potential of smartphones to harbour microorganisms and emphasised the effectiveness of routine device cleaning.

Health promotion messages were delivered through multiple channels. During sample collection sessions, participants received face-to-face education regarding smartphone contamination and were directed to the website through a QR code. After the sample collection, participants were provided with alcohol swabs to encourage immediate adoption of smartphone hygiene practices. Following laboratory analysis, individual MRSS and TVC results, together with educational materials and a link to our website to help participants contextualise the findings, were disseminated via email, enabling participants to reflect on the effectiveness of their existing smartphone hygiene practices.

By 11 August 2026, the educational website had recorded 4348 engagement events and attracted visitors from several countries. However, the effectiveness of the health promotion intervention could not be evaluated, as behavioural or microbiological outcomes were not assessed after the campaign. Therefore, no conclusions can be drawn regarding whether the intervention resulted in changes in participants’ behaviours or smartphone contamination levels.

## 4. Discussion

The median TVC of smartphones sampled in this study was 4 CFU, whereas the mean TVC was 20.6 CFU, indicating a right-skewed distribution in which most devices carried relatively low microbial loads, while a small proportion exhibited substantially higher contamination levels. A similar pattern was observed among healthcare students by Maurici et al. [12], suggesting that smartphone contamination is concentrated in a minority of highly contaminated devices rather than being evenly distributed across devices.

Male students (AOR = 2.497, *p* = 0.025) and students from public universities (AOR = 2.730, *p* = 0.016) had significantly higher odds of high microbial contamination on their smartphones (Table 3). The association with male sex may reflect previously reported differences in hygiene behaviours, particularly lower hand hygiene compliance among men than women [13,14]. Public university students also had greater odds of high microbial contamination on their phones, which may be related to differences in clinical learning environments. In this study, the participating public universities had closer links with large tertiary teaching hospitals, and the affiliated hospitals were located within or near the university campuses, potentially resulting in greater student exposure to clinical environments. Increased contact with patients, shared equipment, and frequently touched environmental surfaces may provide additional opportunities for microbial transfer to smartphones. Healthcare environments are recognised as settings where microorganisms may be transmitted through contact with patients, healthcare workers, contaminated equipment, and environmental surfaces [15]. Nevertheless, university type may also reflect other unmeasured factors, including differences in institutional infection prevention and hygiene practices.

The prevalence of MRSS contamination in the present study was 6.1%, which was lower than that reported in Zambia (25.6% MRSS-positive; 117 healthcare workers) and Pakistan (14.7% MRSA-positive; 259 doctors and clinical-phase medical students), but higher than that reported in a previous Malaysian study at a public university not included in this study (1.2% MRSA-positive, 163 health science students, laboratory staff, and clinical instructors) [5,16,17]. It was also lower than the MRSA prevalence reported among 95 medical students at another private university in Malaysia (9.5%) [18]. Comparisons should therefore be interpreted cautiously because of differences in study populations, clinical exposure, detection methods (the cited studies relied on microbiological identification alone), and target organisms (MRSA, MRSS, or MR-CoNS) investigated. Notably, all MRSS isolates recovered in the present study were MR-CoNS (Table 2). This finding highlights the potential importance of MR-CoNS as reservoirs of antimicrobial resistance on personal devices. Although numerous studies have investigated overall bacterial contamination and MRSA on smartphones, relatively little is known about the species diversity of MR-CoNS colonising these devices. Therefore, the molecular identification of MR-CoNS in the present study provides novel epidemiological data on the diversity of methicillin-resistant staphylococci present on medical students’ smartphones and contributes to the limited literature on this understudied group of organisms.

Living with elderly individuals was significantly associated with MRSS contamination on smartphones (OR = 6.304, *p* = 0.013) (Table S2). Older adults may be more likely to carry multidrug-resistant organisms because of more frequent visits to healthcare facilities, multiple comorbidities, and frequent antibiotic exposure [19]. Consequently, students residing with elderly household members may experience greater opportunities for indirect exposure through close contact and shared living environments. In addition, smartphones with high microbial contamination were more than 12 times as likely to harbour MRSS (OR = 12.194, *p* = 0.001) (Table S2), suggesting that a greater overall microbial burden increases the likelihood of recovering antimicrobial-resistant organisms.

Several variables showed borderline associations. Participants unaware that smartphones can carry microorganisms had lower odds of high microbial contamination (AOR = 0.299, *p* = 0.070) (Table 3). Although this association did not reach statistical significance, the inverse direction of the association was unexpected, as greater awareness would generally be expected to encourage better hygiene practices and, consequently, lower contamination. One possible explanation is that awareness may not accurately reflect actual hygiene behaviour. Participants who were aware that smartphones can harbour microorganisms may have had greater environmental exposure to microorganisms, which could contribute to higher contamination despite greater awareness. Conversely, participants without such awareness may have engaged in hygiene practices that inadvertently reduced microbial contamination. However, this unexpected finding may also reflect residual confounding or a chance observation.

Similarly, placing smartphones on toilet surfaces showed a possible association with MRSS contamination (OR = 6.076, *p* = 0.082) (Table S2), consistent with previous studies identifying toilet-related phone use as a potential source of bacterial contamination [20,21]. Living with healthcare workers also showed a possible association with high microbial contamination (AOR = 1.935, *p* = 0.116) (Table 3), potentially reflecting indirect exposure to healthcare-associated microorganisms in the household environment.

No significant associations were observed between microbial/MRSS contamination and smartphone cleaning frequency (MRSS: OR = 1.305, *p* = 0.744; TVC: OR = 1.591, *p* = 0.261), timing of the last cleaning (MRSS: OR = 0.621, *p* = 0.732; TVC: OR = 1.206, *p* = 0.705), cleaning method (MRSS: OR = 1.861, *p* = 0.500; TVC: OR = 0.864, *p* = 0.711), or recent contact with known MRSS patients (MRSS: OR = 0.897, *p* = 1.000; TVC: OR = 1.531, *p* = 0.404) (Tables S2 and S3). This may be attributable to rapid recontamination of smartphones following cleaning, inconsistent cleaning practices, including alcohol evaporation from opened wipe packs, and limitations inherent in self-reported behavioural data. Furthermore, the small number of MRSS-positive samples limited the statistical power to detect associations. Larger multicentre studies incorporating objective assessments of smartphone hygiene practices are needed to further clarify the determinants of smartphone contamination.

### Limitations

Several limitations should be considered. First, the use of convenience and snowball sampling may have introduced selection bias and limited the generalisability of the findings beyond medical students from selected universities in Klang Valley.

The relatively small number of MRSS-positive isolates (*n* = 9) limited statistical power for analyses involving MRSS contamination and precluded multivariable regression modelling. Consequently, some true associations may not have been detected, and effect estimates should be interpreted with caution.

The cross-sectional design precluded causal inference, as exposures and contamination status were assessed simultaneously. In addition, behavioural data were self-reported and may have been subject to recall and social desirability bias.

Methicillin resistance was determined phenotypically using cefoxitin disc diffusion without molecular confirmation of *mecA* or *mecC*. Although the EUCAST-recommended cefoxitin disc test is widely accepted for detecting methicillin resistance, molecular characterisation would have provided greater insight into the genetic basis of resistance.

Another important limitation of this study was the lack of standardisation of the sampled surface area across smartphones. Although the entire front and back surfaces were sampled, the actual surface area sampled may have varied depending on the size and design of individual smartphones and their covers. This variation may have influenced the number of colonies recovered and, consequently, the comparability of microbial counts between smartphones.

The definition of high microbial contamination was based on the study-specific 75th percentile because no universally accepted microbiological threshold exists for smartphone contamination. Therefore, comparisons with other studies using different definitions should be interpreted cautiously. Given the lack of a standardised threshold, the findings regarding high microbial contamination should be considered exploratory and interpreted primarily within the context of this study.

As participants were recruited from six universities, potential clustering of students within universities may have resulted in correlated observations. Therefore, the observed association between university type (public and private status) and high microbial contamination should be interpreted cautiously, as it may partly reflect university-specific characteristics rather than the public or private status of the institution.

Finally, smartphone contamination was assessed at a single time point, preventing evaluation of temporal changes in microbial carriage. Despite these limitations, the study provides valuable baseline data on smartphone contamination and MRSS carriage among Malaysian medical students.

## 5. Conclusions

This study demonstrated that smartphones used by medical students were frequently contaminated with microorganisms, and a subset also carried MR-CoNS. The presence of MR-CoNS suggests that personal smartphones may serve as reservoirs of antimicrobial-resistant staphylococci and represents a potential infection-control concern, particularly among individuals with clinical exposure.

The finding that all recovered MRSS were MR-CoNS is noteworthy, as information regarding the diversity of MR-CoNS on smartphones remains limited. These organisms are recognised as important reservoirs of antimicrobial resistance genes and potential opportunistic pathogens in both healthcare and community settings, highlighting the potential public health relevance of their presence on personal devices.

However, the cross-sectional design and lack of standardisation of the sampled surface area limit conclusions regarding the extent and direction of microbial transmission. Further multicentre and longitudinal studies are warranted to clarify the persistence, sources, and potential transmission of antimicrobial-resistant staphylococci associated with smartphones.

## Figures and Tables

**Table 1:** Number of sampled medical students from each university.

University	Medical Students		Total
	Preclinical	Clinical	
Public A	12	13	25
Public B	17	6	23
Public C	7	10	17
Private D	9	16	25
Private E	10	11	21
Private F	22	15	37
Total	77	71	148

**Table 2:** Characterisation of presumptive isolates using cefoxitin susceptibility testing and 16S rRNA PCR.

Isolate	Cefoxitin Inhibition Zone Size * (mm)	Interpretation	Species Identity ^#^ (% Query Coverage, % Identity)
P006	14	R	*Staphylococcus haemolyticus* (100, 100)
P039	No zone	R	*Staphylococcus epidermidis* (100, 100)
P052	15	R	*Staphylococcus ureilyticus* (100, 100)
P097	18	R	*Staphylococcus epidermidis* (100, 100)
P099	18	R	*Staphylococcus epidermidis* (100, 100)
P112	No zone	R	*Staphylococcus epidermidis* (100, 100)
P133	17	R	*Staphylococcus haemolyticus* (100, 100)
P145	25	R	*Staphylococcus epidermidis* (100, 100)
P147	No zone	R	*Staphylococcus epidermidis* (100, 100)

* EUCAST 2026 cefoxitin resistance breakpoints [9]: *S. epidermidis* and *S. lugdunensis*, R < 27 mm; *S. aureus* and other CoNS, R < 22 mm; R: resistant; ^#^ BLAST results of 16S rRNA sequencing.

**Table 3:** Univariate analysis and multivariable binary logistic regression for TVC.

Variable	Univariate Analysis		Multivariable Logistic Regression	
	COR (95% CI)	*p*	AOR (95% CI)	*p*
Male sex	2.713 (1.274–5.776)	0.012	2.497 (1.120–5.568)	0.025
Staying with a healthcare worker(s)	2.225 (1.032–4.795)	0.059	1.935 (0.850–4.406)	0.116
Not knowing that the phone can carry microorganisms	0.307 (0.0869–1.086)	0.086	0.299 (0.081–1.102)	0.070
Attending a public university	3.365 (1.551–7.302)	0.002	2.730 (1.206–6.177)	0.016

COR: crude odds ratio; AOR: adjusted odds ratio; CI: confidence interval.

## Data Availability

The original contributions presented in this study are included in the article/Supplementary Materials. Further inquiries can be directed to the corresponding author.

## Supplementary Materials

The following supporting information can be downloaded at https://www.mdpi.com/article/10.3390/antibiotics15100952/s1, Supplementary Material S1: Questionnaire used in this study, Table S1: Descriptive statistics, Table S2: Inferential statistics for MRSS contamination using Fisher’s exact test, Table S3: Inferential statistics for TVC using Fisher’s exact test.

## Abbreviations

The following abbreviations are used in this manuscript:

AMR	Antimicrobial Resistance
AOR	Adjusted Odds Ratio
AST	Antimicrobial Susceptibility Testing
ATCC	American Type Culture Collection
BLAST	Basic Local Alignment Search Tool
CFU	Colony-Forming Units
CI	Confidence Interval
CoNS	Coagulase-Negative Staphylococci
COR	Crude Odds Ratio
EPV	Events Per Variable
EUCAST	European Committee on Antimicrobial Susceptibility Testing
ITS	Internal Transcribed Spacer
MBBS	Bachelor of Medicine, Bachelor of Surgery
MR-CoNS	Methicillin-Resistant Coagulase-Negative Staphylococci
MRSA	Methicillin-Resistant *Staphylococcus aureus*
MRSS	Methicillin-Resistant *Staphylococcus* Species
NCBI	National Center for Biotechnology Information
OR	Odds Ratio
PCR	Polymerase Chain Reaction
rDNA	Ribosomal Deoxyribonucleic Acid
rRNA	Ribosomal Ribonucleic Acid
TVC	Total Viable Count
UTAR	Universiti Tunku Abdul Rahman
WHO	World Health Organization
