# Supplementary material for: Prevalence and Factors Associated with Microbial and Methicillin-Resistant Staphylococcal Contamination of Smartphones Among Medical Students at Six Universities in Klang Valley, Malaysia
Source: Antibiotics (Basel). 2026 Sep 24;15(10):952. doi: 10.3390/antibiotics15100952 (PMC13700749; doi:10.3390/antibiotics15100952)
Supplement: Supplementary file 1 [file antibiotics-15-00952-s001.zip › Supplementary Material S1.pdf]

Supplementary Material S1. Questionnaire used in this study.

Demographic and General Information:

1. Full name
2. Phone number
3. Email address (for result dissemination purpose)
4. Age
5. Sex
    a. Female
    b. Male
6. Ethnicity
    a. Bumiputera (indigenous)
    b. Chinese
    c. India
    d. Malay
    e. Other:
7. University (this is anonymised as A—F in the publication)
    a. A
    b. B
    c. C
    d. D
    e. E
    f. F
8. Year of study
    a. Clinical year (Year 3 - Year 5)
    b. Pre-clinical year (Year 1 - Year 2)
9. Do you think your phone can carry harmful microorganisms?
    a. No
    b. Yes

10. Do you currently have any skin infections or wounds?

 a. No

 b. Yes

Behavioural Factors

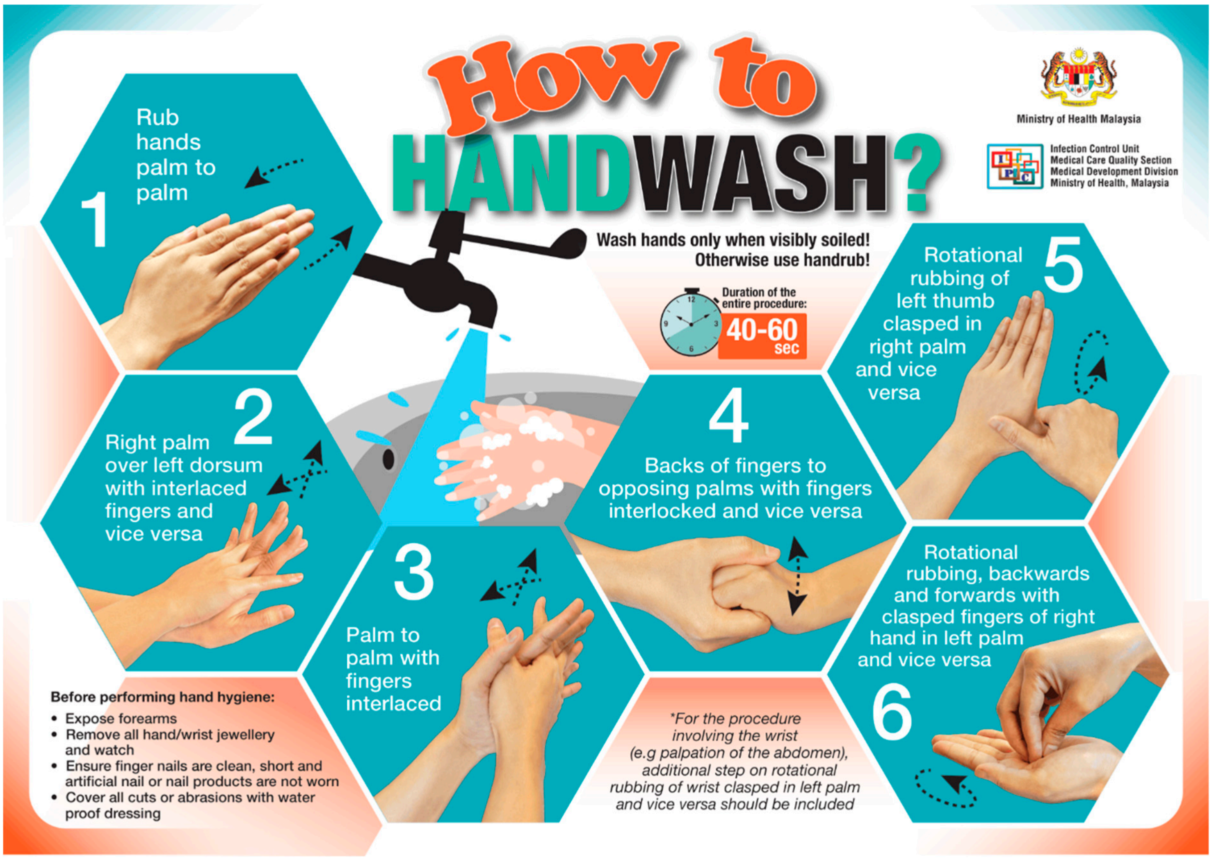

1. Do you wash your hands following Ministry of Health (MOH) Malaysia guidelines?

 a. No

 b. Yes

2. Do you sanitise your hands with hand sanitiser?

 a. No

 b. Yes

3. Do you wash your hands after toilet use?

 a. No

 b. Yes

4. Do you wash your hands with soap after toilet use?

    a. No
    b. Yes

5. Do you use your phone in the toilet?
    a. No
    b. Yes
6. Do you put your phone on any surfaces in the toilet (e.g., countertop or shelf of a basin or toilet)?
    a. No
    b. Yes
7. Do you share your phone with others?
    a. No
    b. Yes
8. How frequently do you clean your phone?
    a. Daily
    b. At least once a week
    c. At least once a month
    d. At least once a year
    e. Never
9. Do you have any history of antibiotic use within the past 3 months?
    a. No
    b. Yes
10. Do you play contact sports (e.g., basketball, martial art)?
    a. No
    b. Yes
11. Do you use your phone in crowded areas (e.g., public transport, concert, night market)?
    a. No
    b. Yes

Behavioural Factors (Phone Hygiene)

*The participants will skip this section if they never cleaned their phones before (in the previous section)

1. When was the last time you cleaned your phone? (Choose the most relevant option)
   a. Within the last 24 hours
   b. Within the last 7 days
   c. Within the last 30 days
   d. Within this year
2. What is your phone-cleaning method?
   a. Antibacterial wet tissue/ alcohol-based disinfectants
   b. Normal wet wipes (non-bacterial)
   c. Dry tissue paper only

Environmental Factors

1. Do you have a history of hospital admission for more than 1 day within the past 6 months?
   a. No
   b. Yes
2. Do you spend at least 4 hours in the last seven days staying with an elderly person (aged $\geq$ 60) (e.g., weekends at home with your grandparents)?
   a. No
   b. Yes
3. Do you spend at least 4 hours in the last seven days staying with anyone working in the hospital/ healthcare setting?
   a. No
   b. Yes

Behavioural Factors (Clinical-Year Students Only)

1. Do you use your phone during ward rounds?
    a. No
    b. Yes
2. Do you use your phone during patient examination?
    a. No
    b. Yes
3. Do you use your phone in between patient examinations?
    a. No
    b. Yes
4. Do you wash your hands or clean your hands with sanitiser after patient contact?
    a. No
    b. Yes
5. Do you use your phone with gloves on during posting?
    a. No
    b. Yes
6. Do you place your phone on hospital surfaces (e.g., beds, trolleys, desks)?
    a. No
    b. Yes

Environmental Factors (Clinical-Year Students Only)

1. Which hospital department are you currently posted in?
    a. Anesthesiology/ Critical Care/ ICU
    b. Dermatology
    c. Ear, Nose and Throat (ENT) or Otorhinolaryngology
    d. Emergency Medicine
    e. Family Medicine/ Primary Care/ Klinik Kesihatan
    f. Forensic Medicine
    g. General Surgery
    h. Internal Medicine
    i. Obstetrics and Gynaecology (O&G)
    j. Ophthalmology
    k. Orthopaedics
    l. Pharmacology/ Clinical Pharmacy
    m. Pathology/ Laboratory Medicine
    n. Paediatrics
    o. Psychiatry
    p. Public Health
    q. Radiology
    r. Rehabilitation
    s. Other:
2. Please indicate the clinical posting you attended prior to your current posting.
    a. Anesthesiology/ Critical Care/ ICU
    b. Dermatology
    c. Ear, Nose and Throat (ENT) or Otorhinolaryngology
    d. Emergency Medicine
    e. Family Medicine/ Primary Care/ Klinik Kesihatan
    f. Forensic Medicine
    g. General Surgery

h. Internal Medicine
i. Obstetrics and Gynaecology (O&G)
j. Ophthalmology
k. Orthopaedics
l. Pharmacology/ Clinical Pharmacy
m. Pathology/ Laboratory Medicine
n. Paediatrics
o. Psychiatry
p. Public Health
q. Radiology
r. Rehabilitation
s. None (I'm currently in my first posting)
t. Other:

3. Have you had contact with wound-care patients within the recent postings (i.e., current and immediate-past postings)?
    a. No
    b. Yes
4. Have you had contact with ICU patients within recent postings (i.e., current and immediate-past postings)?
    a. No
    b. Yes
5. Have you had contact with patients with known methicillin-resistant *Staphylococcus* infections within recent postings (i.e., current and immediate-past postings)?
    a. No
    b. Yes
